# A Method for Generating Real-Time Indoor Illuminance Maps Based on Images in a Sensorless Lighting Environment

**DOI:** 10.3390/s26165261

**Published:** 2026-08-19

**Authors:** Seung-Teak Oh, You-Bin Lee, Jae-Hyun Lim

**Affiliations:** 1 Smart Natural Space Research Center, Kongju National University, Cheonan 31080, Republic of Korea; ost73@kongju.ac.kr; 2 Department of Computer Science & Engineering, Kongju National University, Cheonan 31080, Republic of Korea; youbbqlsl@smail.kongju.ac.kr

**Keywords:** illuminance map, sensorless, lighting, image, generating indoor illuminance map

## Abstract

In the field of system lighting control, which aims to improve light quality and reduce energy consumption, analyzing indoor illuminance is an essential technology. Researchers have collected and analyzed illuminance data using multiple light sensors in a room. However, operating various sensing devices and related equipment incurs additional maintenance costs and causes user inconvenience, thereby hindering the widespread adoption of such system lighting technologies. Achieving a higher level of natural light-integrated lighting technology requires methods for analyzing indoor illuminance with minimal intervention from physical sensors, yet research in this area has been severely lacking. Therefore, this study proposes a method for generating image-based indoor illuminance maps in a sensorless lighting environment. Thus, an indoor light environment dataset was constructed by collecting illuminance and floor images from various zones in an experimental environment with natural light. Through dataset-based analysis, color elements in images that were highly correlated with illuminance were identified, and a CNN-based deep learning model was trained to estimate illuminance at each point using indoor images as input. A general method for calculating real-time illuminance based on an indoor image has been proposed. In experiments, this method demonstrated a Mean Absolute Percentage Error (MAPE) of within 6% for individual points during daytime hours, and 11% daily, even when indoor illuminance fluctuated significantly.

## 1. Introduction

Indoor illumination is a key factor in improving the visual comfort and work productivity of building occupants [1]. Efficient management of indoor lighting is crucial in smart building management systems, which aim to achieve energy savings [2,3]. Recently, with the development of LED lighting and IoT-related technologies, lighting control systems utilizing natural light have gained attention [4,5]. Attempts have been made to apply system lighting technology to the indoor light environment formed by natural light inflowing through windows and artificial lighting, thereby providing beneficial lighting while saving energy [6,7]. However, unlike controllable artificial lighting, natural light varies constantly, producing unpredictable intensity distributions across different areas of a room [8,9]. Therefore, implementing a lighting system integrated with natural light requires technology capable of continuously monitoring changing indoor light levels in real time [8,10]. A standard method for monitoring indoor light levels involves installing multiple sensors at multiple points in the room to collect and analyze light-intensity data [9,11]. However, operating multiple sensors entails costs for installing and maintaining sensing and communication equipment. Also, it raises technical issues, such as increased power consumption during sensor network operation and delays, and losses in the transmission of sensing data [12,13]. Furthermore, the space required for installing multiple sensors impairs user convenience and comfort [11,13]. The challenges of operating various sensors limit the implementation and commercialization of lighting technologies that use natural light [14]. Previous studies have introduced different methods to address these problems. These methods include calculating illuminance across the entire room using a single sensor installed near a window or analyzing the indoor lighting environment using simulation software [15,16]. Bouroussis et al. proposed an analytical formula to compute illuminance at multiple indoor points from ceiling-mounted images captured with a camera [17,18]. However, when the minimum number of sensors is used, it is difficult to position the sensors and to guarantee indoor illuminance prediction, especially when there are significant variations in natural light inflow characteristics. Image-based methods were unable to completely capture the variations in lighting caused by natural light [19]. The illuminance of the work plane was predicted using a machine learning-based model incorporating DSLR cameras and illuminance meters. However, this approach did not account for the actual lighting conditions throughout the entire indoor space [20]. Most existing image-based techniques for predicting illuminance primarily focus on identifying elements within images, with few studies calculating detailed indoor illuminance levels for integration with lighting systems [21]. For instance, significant changes in illumination at specific points were observed. Furthermore, simulation software-based methods required a variety of complex input parameters, such as the building’s location and wall materials. These methods also struggled to accurately reflect the real-time changes in natural light [22].

Recently, various attempts have been made to predict indoor illuminance levels by applying deep learning-based intelligent prediction models and integrating natural lighting systems [23,24]. Ngarambe et al. predicted daylight illuminance using simulation data and various machine learning algorithms, such as DNNs and LSTMs. They also compared the performance of each method [25]. Velez et al. created a virtual indoor environment with adjustable lighting settings and proposed a deep learning-based approach to estimate illuminance using feedforward neural networks and convolutional neural networks [26]. In a previous study, an approach was introduced for acquiring RGB images using ceiling-mounted cameras and estimating illuminance using a deep learning model. The illuminance values at selected reference points were then estimated for spatial interpolation, and these values were used to generate a detailed illuminance map using the Kriging technique [27]. Seniguer et al. aimed to predict illuminance levels for various zones from indoor images captured using smartphone cameras. They also compared the accuracy of different models, including Random Forest [28]. In addition, illuminance at specific points was predicted using a CNN-MLP model after acquiring image of window scenes, which acted as side light sources, and changes in indoor lighting [29]. However, these methods have limitations: they require complex inputs, such as weather data and spatial configurations, or they remain at an experimental stage, which is insufficient to generate detailed indoor illuminance maps. Even when combining image analysis with deep learning, achieving true real-time illuminance calculation remains impossible. Furthermore, due to constraints such as unstable accuracy based on weather conditions and the use of limited natural light datasets, these approaches were not fully realized [25]. Therefore, despite these various efforts, accurately calculating indoor illuminance in the presence of natural light remains difficult. Research on sensorless lighting systems that automatically analyze illuminance across all areas of an indoor environment and control lighting accordingly, without user intervention, in environments created by both natural and artificial light, remains in its preliminary stages. Furthermore, research on eliminating the physical sensors necessary for the widespread adoption of such lighting technology has been severely lacking.

This paper proposes a method for generating an image-based indoor illuminance map that enables easy monitoring of illuminance levels across all areas, even in environments without optical sensors. To achieve this, a pre-experimental indoor environment with natural light was established, and illuminance data at multiple points, along with floor images directly below the ceiling reference point, were collected to create an indoor lighting dataset. Subsequently, correlations among dataset elements were analyzed to identify image color features or models that were strongly correlated with illuminance. Based on this, a CNN-based deep learning model that took indoor images as input and computed illuminance at each point was developed. A detailed indoor illuminance map was then generated by calculating and combining illuminance values at each point in the indoor space, which varied continuously with incoming natural light. Furthermore, by evaluating performance using images captured at one-minute intervals under actual natural lighting conditions, we propose a method to create a sensorless lighting environment suitable for general application.

## 2. Collection and Analysis of Indoor Lighting Environment Properties

This paper proposes a method for accurately determining the illuminance in all areas of an indoor environment without the use of illuminance sensors. It was necessary to analyze the relationships among the factors influencing the creation of the light environment in ordinary indoor spaces, where natural light exhibited significant variations. Therefore, an experimental environment was constructed to collect images of the indoor space and illuminance data at multiple points over time. The feasibility of the proposed method was verified by collecting and analyzing characteristics of the indoor light environment, and a dataset was created for training a deep learning model to generate an indoor illuminance map from images.

**Figure 1:**
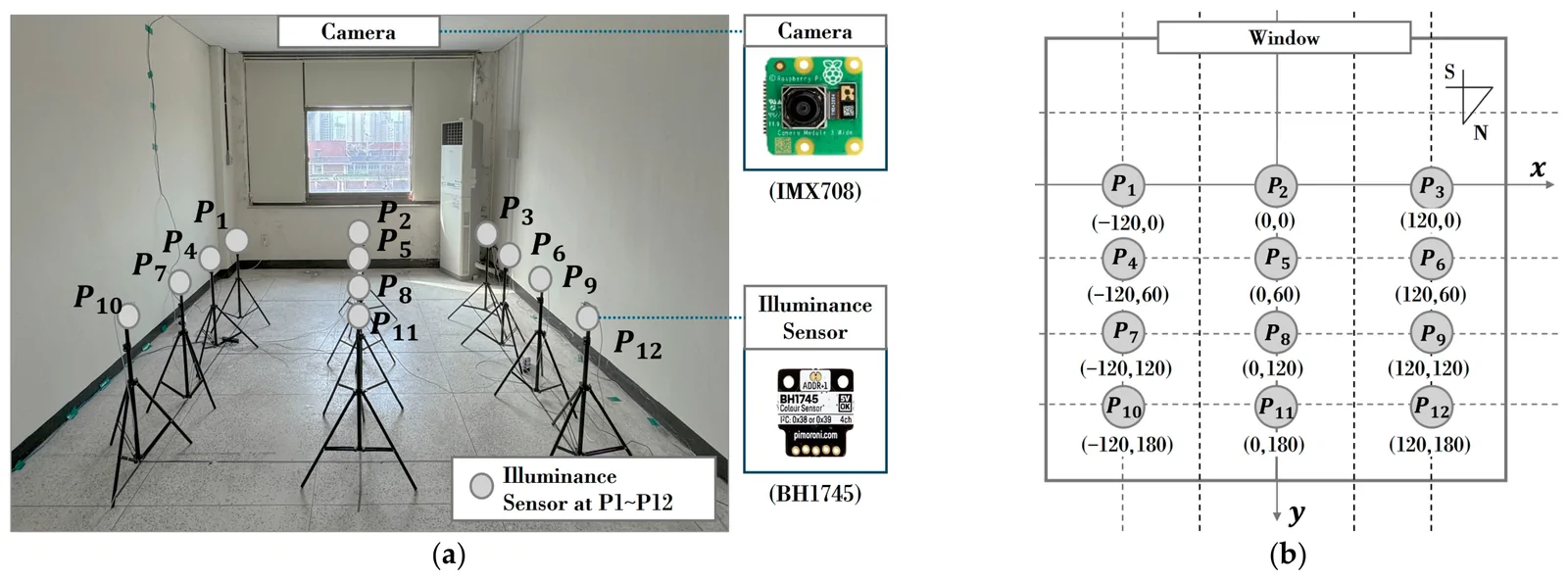
Data collection environment for indoor light environment characteristics. (**a**) Experimental setup: Camera and illuminance sensor. (**b**) Overview of the experimental environment and location classification.

To analyze changes in illuminance across points in the room due to the influx of natural light and to obtain the dataset necessary to build a deep learning model that generates an image-based illuminance map, an experimental environment was established. The experimental environment was a laboratory on the fourth floor of a building at K University (Republic of Korea), located at a latitude of 36.85 and a longitude of 127.15. The laboratory space measured 367 cm in width, 500 cm in length, and 264 cm in height, and had one south-facing window measuring 144 cm by 102 cm. A camera module was installed at the center of the ceiling to measure the indoor floor surface. Based on the illuminance measurement method stipulated by the Korean National Standards (KS C 7612) [30], the indoor space was divided into 12 equally spaced points (P1–P12), and light characteristic sensing devices were installed at each point. Figure 1 shows the results of the experimental environment setup.

**Figure 2:**
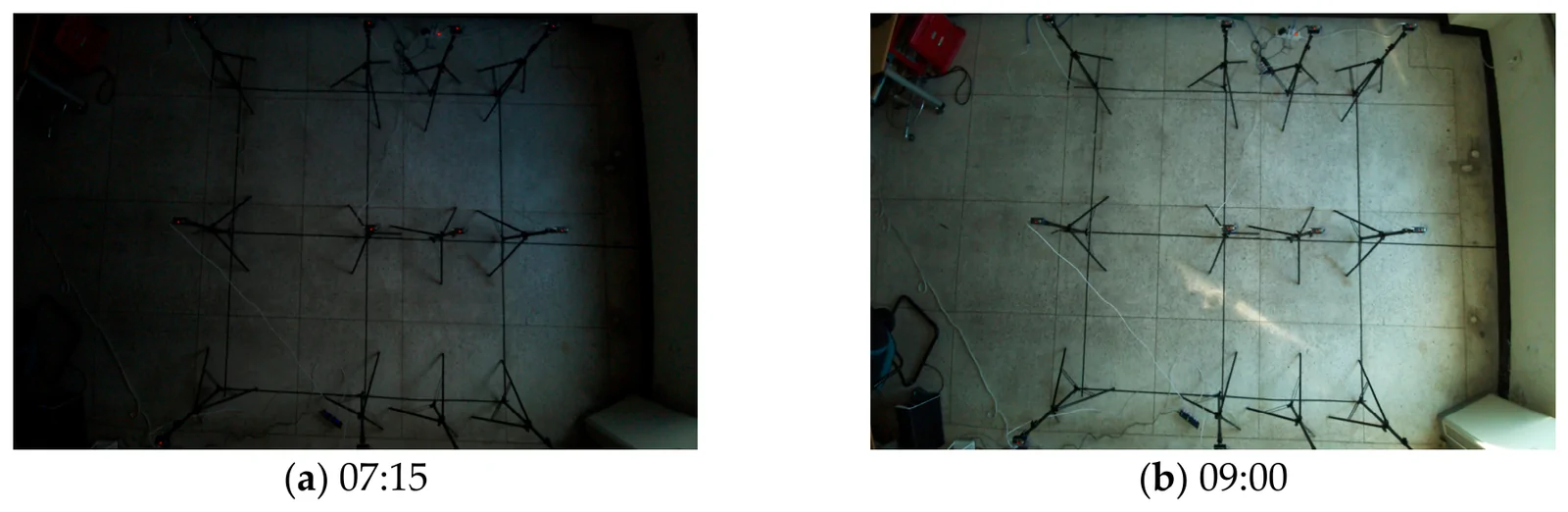

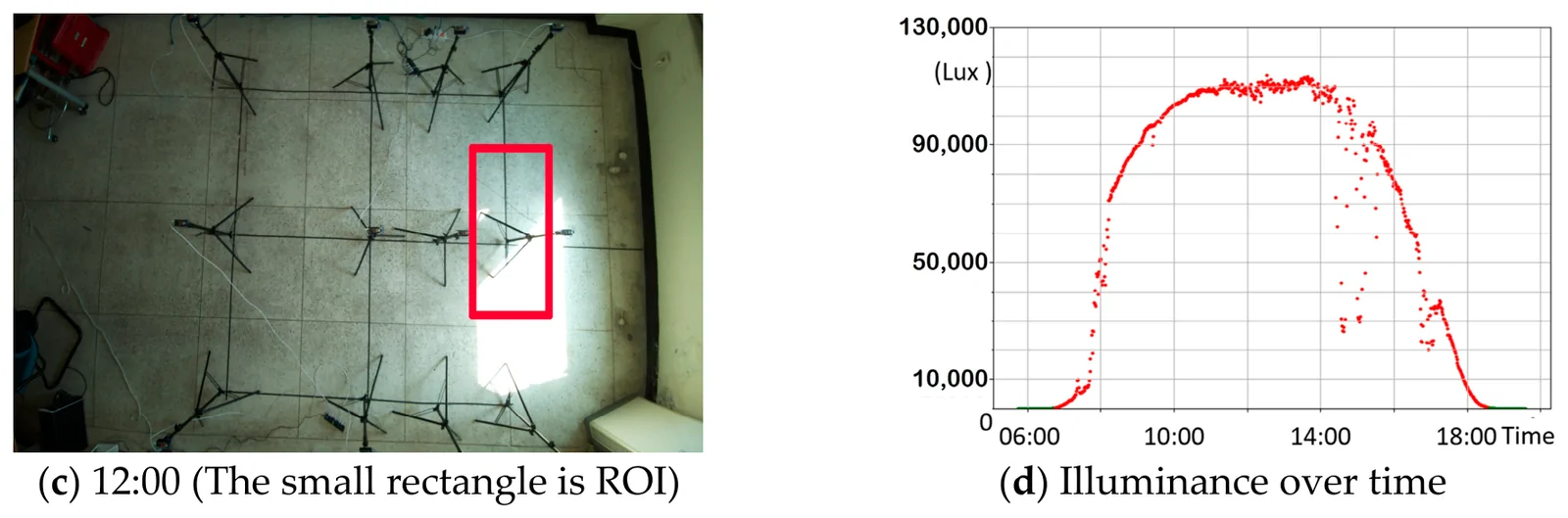
Measurement examples of floor surface image and outdoor illuminance.

As shown in Figure 1a, for the ceiling-mounted camera (hereinafter referred to as the ceiling camera), a Raspberry Pi Camera Module 3 Wide Module (Arducam, Hong Kong, China) was used. It was equipped with a Sony IMX708 sensor (Sony Corporation, Tokyo, Japan) and offers a 120-degree field of view. The ceiling camera’s exposure time was fixed at 10,000 μs, thereby maintaining image brightness despite changes in indoor lighting conditions. The camera’s gain and white balance properties were set to auto mode to ensure the use of the standard camera settings. In addition, as shown in Figure 1b, sensors capable of measuring illuminance (BH1745, Pimoroni, Sheffield, UK) were installed at 12 points (P1–P12) inside the room. Additionally, as shown in (b), RGB sensors Pimoroni, Sheffield, UK) capable of measuring illuminance were installed at 12 locations within the room. Sensing devices were fabricated by mounting the BH1745 sensor onto a development board equipped with a Wi-Fi module (ESP32-S3-DevKitC-1, Espressif Systems, Shanghai, China) and implementing real-time sensing, data storage, and illuminance calculation functions based on tristimulus values. An optical diffuser was also applied to the sensor’s light receptor to prevent saturation from natural light and minimize measurement errors caused by changes in the direction of incident light. The fabricated sensing devices were calibrated using a spectroradiometer (CAS 140CT, Instrument Systems, Munich, Germany). It was confirmed that they could measure illuminance with an error rate of less than 0.5% under conditions of 1000 lux or lower. The sensor was mounted 80 cm above the floor, consistent with typical desk height in a general office environment. To facilitate easy analysis of the measured illuminance values at each point, the central point near the window (P2) was set as the origin (0, 0), and the coordinates of each point were defined using the X and Y distances from the origin. The ceiling cameras and illuminance sensors, as well as their functionality, were implemented to collect sensing data in a wireless communication environment. Over approximately six months (7 March 2024, to 1 September 2024), images of the floor surface and illuminance measurements at various points were collected at 1 min intervals, yielding an indoor lighting environment dataset, as shown in Table 1. The indoor lighting environment dataset includes a total of 106,651 records. Figure 2 presents an example of floor surface images taken on 13 March 2024. To compare and verify the external natural light conditions on that day, the hourly illuminance measured at the same point using a spectroradiometer (CAS 140CT, Instruments, Germany) was also presented.

**Figure 3:**
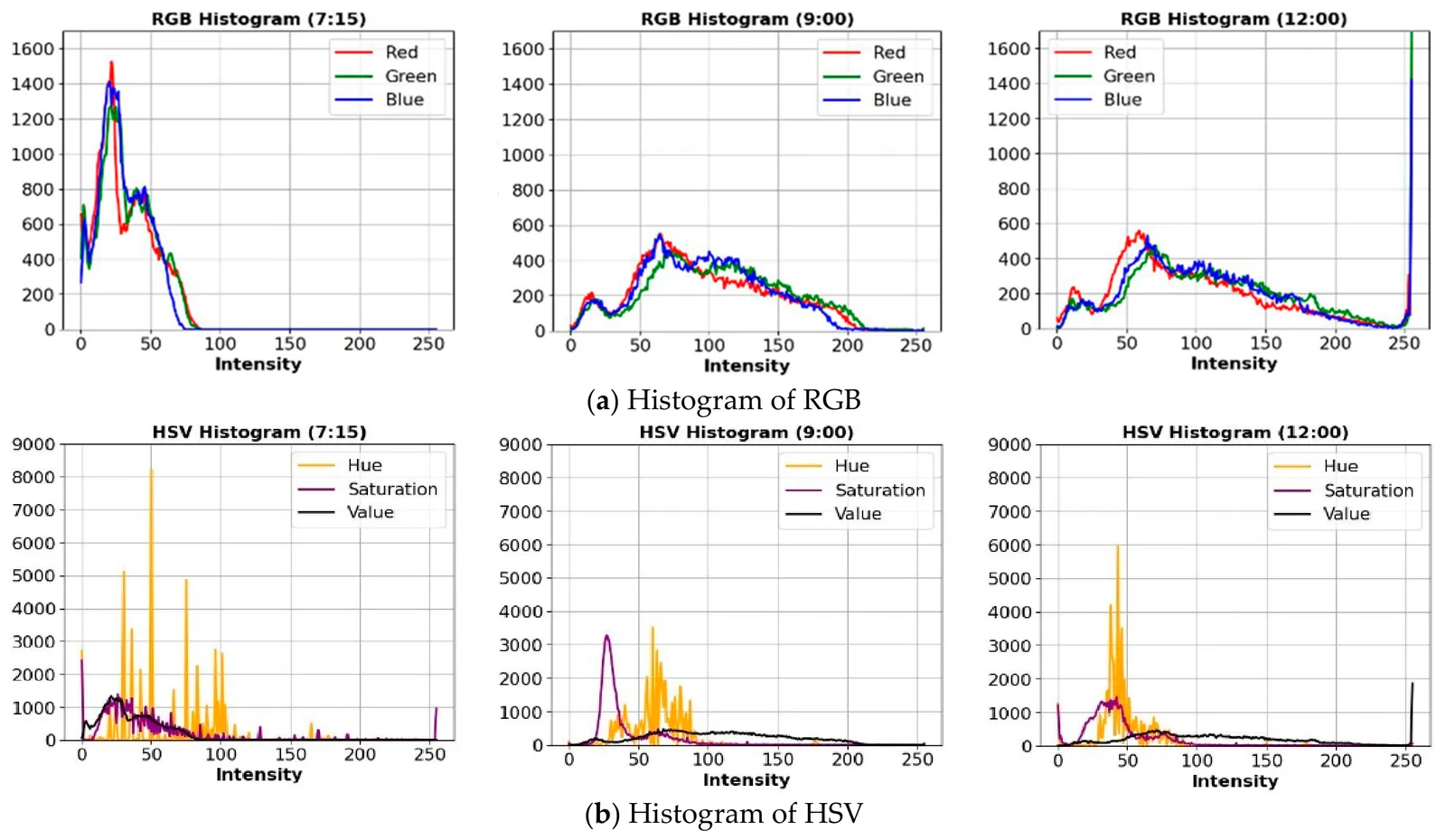
Histograms of indoor floor surface images by color model by hour.

As shown in Table 1, the indoor light environment was created which was dark at close to sunrise (07:15) and bright at 09:00 and 12:00. Due to the changes in natural light over time, an illuminance was unevenly distributed, resulting in brighter or darker areas on the left or right sides of points P1, P5, P8 and P11, located at vertical center relative to the window. These changes were also observed in the indoor images, suggesting a strong correlation between the floor-surface image and illuminance. To confirm this, an analysis was performed to examine the relationship between the characteristics of the image in the middle area near the window, which was significantly affected by natural light, and the measured illuminance at that point (P2). A specific area of the image, including point P2 and located at the center of the window side, was set as the Region of Interest (ROI). The small rectangular area shown in Figure 2c indicates the ROI. The rectangular ROI and color components, such as RGB and HSV, were extracted, and their correlations with illuminance over time (shown in [d]) were investigated. The day in question (13 March 2024) was bright, with an illumination pattern exhibiting a consistent parabolic shape, as shown in Figure 2d. However, there were periods of cloudy or overcast conditions in the afternoon. For the correlation analysis, 735 images of the indoor floor surface, taken at one-minute intervals from sunrise to sunset, were adopted. Indoor floor images for 30 min after sunrise (07:15), 10:00 AM, and noon (12:00) on that day are shown in Figure 2, and the average indoor illuminance levels at each time were 64, 427, and 1263 Lux, respectively. First, indoor floor images at each time point and histograms for each color model were analyzed to identify changes in color-space characteristics with respect to variations in natural light. The color models considered were RGB, Grayscale, HSV, LAB (CIE L*a*b*), and HSL. Figure 3 shows the time-dependent histograms for the RGB and HSV color models.

As shown in Figure 3, the histograms of the individual color models also exhibited distinct patterns with respect to hourly changes in illuminance. In the case of the RGB color model in (a), during sunrise and sunset, when the influx of natural light was low with a low illuminance, the intensity of each RGB channel was low, ranging from 0 to 80. At 09:00, when the natural light gradually brightened, and at noon (12:00), when the sun’s altitude was highest, the intensity values of each channel were evenly distributed in the 0–230 range, with specific values even exceeding 250. Figure 3b shows the case of the HSV color model, where each channel of hue (H), saturation (S), and value (V) changes independently. During low-light periods at sunrise and sunset, the components of the hue channel were observed to be widely dispersed and irregularly distributed. Still, the variation decreased at 09:00 and 12:00. The saturation (S) and value (V) channels showed similar patterns at 07:15 and 18:06, but exhibited different distributions at 09:00 and 12:00. The distribution patterns of the saturation (S) and value (V) channels varied between 09:00 and 12:00, while the patterns observed at 07:15 (early morning) and 18:00 (evening) were similar. Since the distribution pattern at 18:00 resembled that of 07:15, it has been omitted from Figure 3.

As shown in Figure 3, the responses of the color models to changes in timewise illuminance differed. Still, the channel values of each color model in the floor image exhibited a consistent pattern as illuminance varied. Comparable results and patterns were observed in the histogram analysis of the Grayscale, LAB, and HSL color models. After confirming the correlation between indoor illuminance and the channel values of each color model, a correlation analysis was performed to identify which color model attributes were most closely associated with illuminance and, therefore, suitable as input to the proposed method. The average values for the characteristics of the RGB, Grayscale, HSV, LAB (CIE L*a*b*), and HSL color models were calculated for the ROI as shown in Figure 2c. Subsequently, a correlation analysis was performed between the attribute values of each color model and the illuminance at point P2; the results are shown in Table 2.

As shown in Table 2, all elements except for B in the RGB model, S in the HSV model, and L in the HSL model showed positive and negative correlations of ±0.6 or higher. In particular, H in the HSV model, B in the LAB model, and H in the HSL model showed strong correlations of ±0.86 or higher. This confirms that most color attributes derived from the floor image exhibit significant correlations with illuminance. Furthermore, by selecting an appropriate color model and using it as an input dataset for a deep learning model, it would be possible to calculate the illuminance at various points in the indoor environment.

## 3. Image-Based Illuminance Map Generation Method

**Figure 4:**
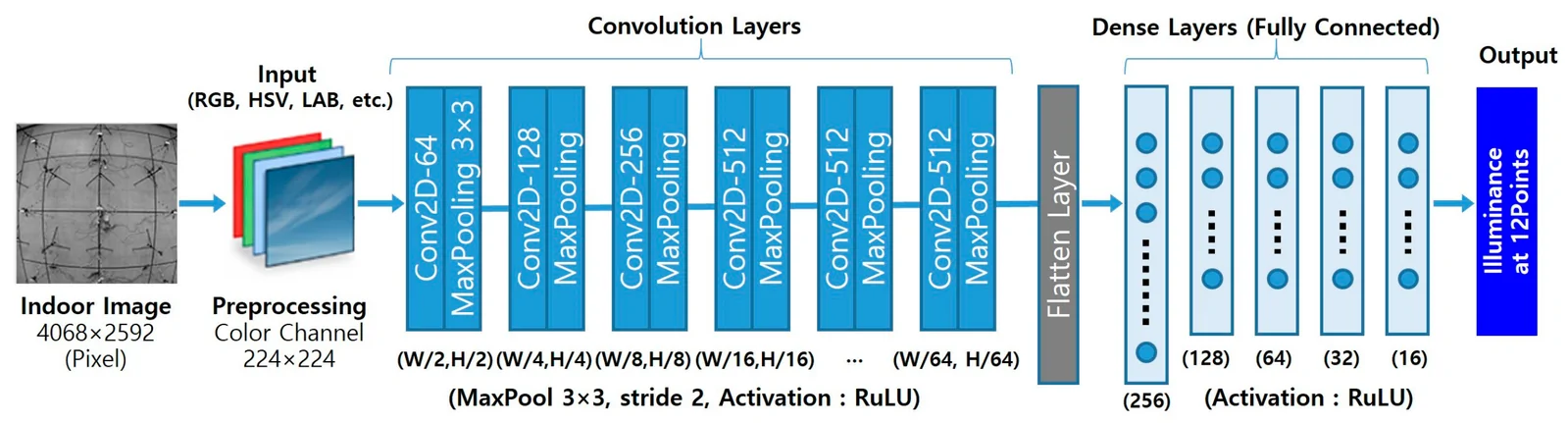
Basic design of the CNN model for calculating illuminance at each point based on floor surface images.

This study proposes a method for determining illuminance across all areas of an indoor space without installing light-measurement sensors in the user’s activity area. A deep learning model was developed and applied to generate a detailed indoor illuminance map using ceiling-mounted floor images as inputs. A CNN model, which is widely used and has superior performance, was applied as a deep learning model for feature extraction or result generation based on images [25,26]. A Convolutional Neural Network (CNN), commonly used to extract features or generate results from images, was employed. While CNN models often use RGB images as input, recent studies have shown improved performance by incorporating attribute values from color models such as HSV and LAB. Therefore, in this research, the basic structure of a CNN model for generating illuminance at each point in an indoor environment was designed; the optimal color model was selected and implemented as the input image; and an optimization process was then applied to improve the performance of the model in generating an image-based illuminance map. Figure 4 shows the basic design of the proposed model, which calculates the illuminance at each point.

As shown in Figure 4, the proposed model takes an image of the indoor floor surface as input and gives outputs of the illuminance at various points within the space. The model was implemented using a PyTorch (2.4.1) framework after establishing an Anaconda (Python 3.11) virtual environment on a Windows 10 Pro PC equipped with an AMD Ryzen 5 5600X processor and an NVIDIA GeForce GTX 1650 graphics card. The input image is a color image with a resolution of 4608 × 2592 pixels. It was resized to 224 × 224 pixels for input. An image normalization process was performed using the OpenCV library to convert the image into various color spaces. The resulting input image passed through six convolutional layers and five fully connected layers. Each convolutional layer employed 3 × 3 filters and the ReLU activation function, sequentially generating 64, 128, 256, 512, 512, and 512 feature maps. Max pooling layers were placed between the convolutional layers to reduce the size of the feature maps, and a flatten layer converted them into a 256-dimensional vector. Finally, the data passed through dense layers with 256, 128, 64, 32, and 16 neurons to produce illuminance values for 12 points. First, to select the most suitable color model for the proposed model’s input, the illuminance prediction performance was compared across various combinations of color models. The training was performed using images from each attribute channel of the RGB, Grayscale, HSV, and LAB color models as input. For training, the dataset excluding the test set was used during the period from 29 March 2024, to 1 September 2024. Daily data corresponding to 20% of the total was randomly selected and set aside as validation data. A single bright day, 7 April 2024, was selected as the test data, resulting in a total dataset of 751 entries. During model training, the mean absolute error (MAE) was used as the loss function, and the Adam optimizer was employed. The number of training frequencies was fixed at 10 for all models. After model training, the quantitative evaluation of each application result used mean squared error (MSE), MAE, and the coefficient of determination (R^2^) as performance indicators. Table 3 presents the illuminance results obtained by the CNN model using various color models as inputs.

As shown in Table 3, a comparison of performance across color spaces indicated that using the HSV color space as the input dataset yielded the best performance. For the HSV color space, the test-day results were an RMSE of 20.948 Lux, an MAE of 15.030 Lux, and an R^2^ of 0.99. In other words, when comparing RMSE and MAE, the error in measured illuminance was small, and the correlation coefficient (R^2^) was high, enabling a more stable calculation of illuminance. As indicated in Table 2 of Chapter 2, the H component demonstrated a very strong negative correlation with illuminance. The S and V components also exhibited significant correlations. Therefore, these factors may have influenced the accuracy of illuminance calculations. Based on this performance comparison, the HSV color model was selected as the optimal input dataset for the CNN to predict illuminance at multiple indoor locations. Subsequently, an optimization process was conducted on the CNN model to predict indoor illuminance at separate locations. The performance comparison was performed for CNN model under each condition, while the number of convolutional layers adjusted from 2 to 6, the number of filters in each convolutional layer to 64, 128, 256, and 512, the number of dense layers from 2 to 5, and the number of nodes in each dense layer to 32, 64, 96, 128, and 256. The optimization experiment results are shown in Table 4, which presents the top 5 conditions exhibiting optimal performance.

**Figure 5:**
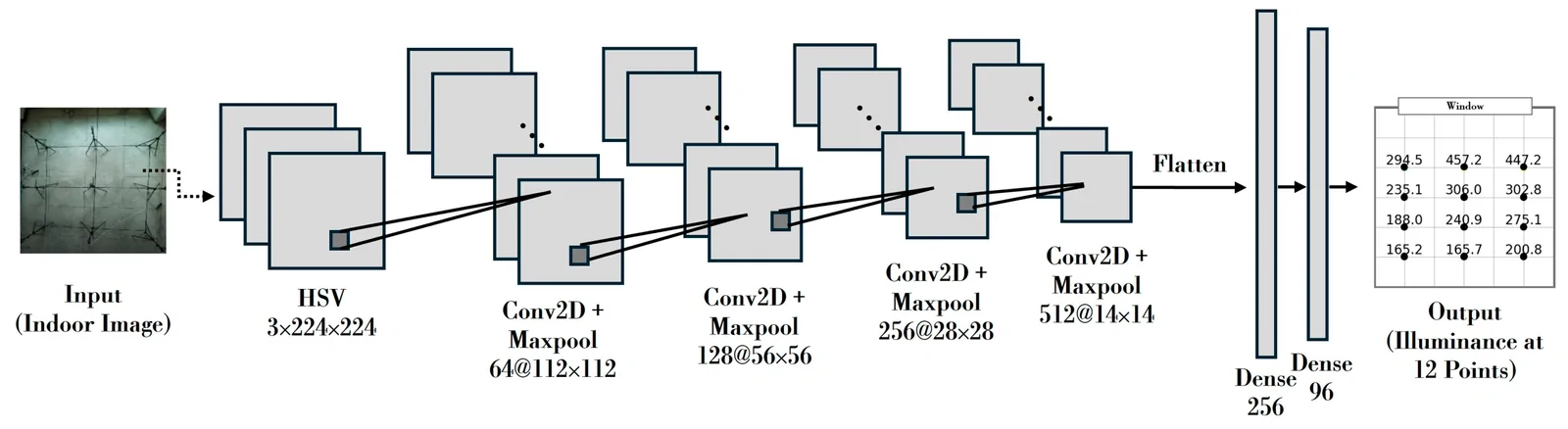
Deep learning model for optimizing illuminance calculation.

Table 4 shows the ranking based on the lowest MAE. Hyperparameter tuning of the proposed model showed that the highest ranking was achieved with four convolutional layers and two fully connected layers. Under these conditions, setting the number of filters in each convolutional layer to 64, 128, 256, and 512, and the fully connected layers to 256 and 96 units, resulted in the lowest MAE of 13.53 Lux, displaying improved accuracy in illuminance calculation compared to the pre-optimization model (MAE of 15.03 Lux). Therefore, a deep learning model for calculating indoor illuminance at specific points was constructed using channel-wise images in the HSV color space as input and configured according to the Rank 1 conditions shown in Table 4. The results are shown in Figure 5.

The proposed model, as illustrated in Figure 5, was designed to output illuminance values for 12 points using four convolutional layers (Conv2D) and two dense layers. The model employed the Adam optimizer with a batch size of 4, MAE as the loss function, a total of 10 epochs, a learning rate of 0.001, and the ReLU activation function. Using the deep learning model shown in Figure 5, illuminance at each point from P1 to P12 was computed, and the results were reconstructed to generate a detailed indoor illuminance map. The proposed method was capable of generating detailed illuminance values by adjusting the number and locations of points. It enabled the generation of an illuminance map using a spatial interpolation method [27].

## 4. Experiments and Analysis

**Figure 6:**
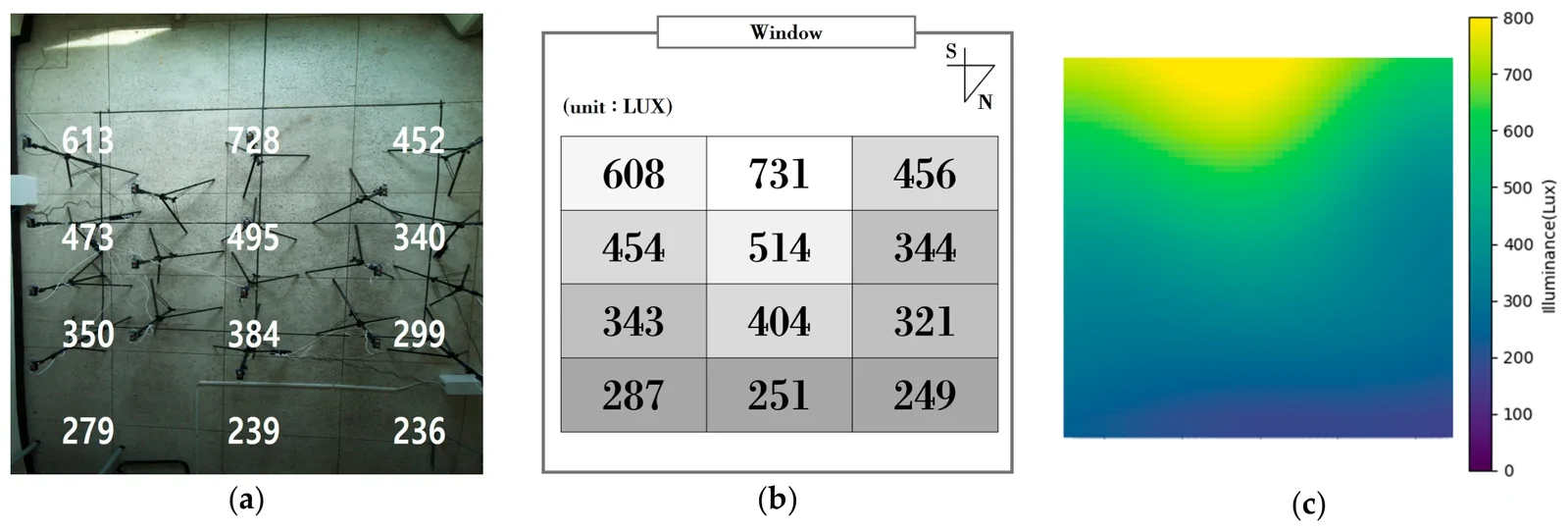
Application results of the proposed method (21 August 2024, 15:21). (**a**) Measured illuminance. (**b**) Illuminance calculated using the proposed method. (**c**) Detailed Illuminance map.

This paper presents an experimental evaluation of the performance of the proposed image-based method for generating indoor illuminance maps. The training dataset for the deep learning model that estimated illuminance at multiple indoor points was constructed from floor images and illuminance data at 12 points within the experimental space. The proposed method was then applied to an arbitrary day that was not included in the training data. The selected day (21 August 2024) exhibited varying natural light conditions, with cloudy skies in the morning and clear skies in the afternoon, thereby allowing the evaluation of performance under diverse weather conditions. Results from 9:00 AM, 1:00 PM, 3:21 PM (when the illuminance at the central window-side point [P2] was maximum), and 6:00 PM (near sunset) were selected for analysis. Figure 6 shows examples of the generated 4 × 3 illuminance maps using indoor floor images from these time periods as input.

Figure 6a shows the measured illuminance values at 12 points indoors, and Figure 6b shows the illuminance map generated by the proposed method using an image of the floor surface as input. Additionally, part (c) provides an example of a detailed illuminance map generated using the spatial interpolation method introduced in a previous study [23]. The proposed method enabled real-time generation of an illuminance map, as shown in Figure 6, allowing users to assess illumination at specific points in a sensorless environment. Illuminance values were calculated for each point at four specific time periods selected considering changes in sunlight, and these values were compared to the measured illuminance at those points. Table 5 presents the results of applying the proposed method for each time period under varying natural light conditions.

As shown in Table 5, the experimental space exhibited significant differences in illuminance across time and areas, attributable to natural light. Based on the measured values, the maximum and minimum illuminance at each point in the room varied: 21–65 Lux at 09:00, 138–388 Lux at 13:00, 236–728 Lux at 15:21, and 72–169 Lux at 18:00. Even at the same time period, the illuminance in different areas of the room varied by 44–492 Lux, confirming the need for detailed indoor illuminance maps when controlling lighting in conjunction with natural light. The proposed method was then applied in this experimental environment, in which the illuminance distribution varied continuously with time and location. As a result, it was possible to generate indoor illuminance maps with values similar to the measured values, with MAE values of 7, 14, 22, and 11 Lux for each time period. Overall, the measured and calculated illuminance values were in close agreement, with a MAPE of 11%. When examined over time, differences in illuminance at each point were not significant during the low-light periods (09:00 and 18:00), and the calculated illuminance differences were also minor during these periods. However, at 13:00 and 15:21, when the indoor environment was bright and there was a significant illuminance imbalance across areas, accurate illuminance values could be calculated with a MAPE of 6%. The performance of the illuminance map calculation was superior during daytime hours when significant variations in indoor illuminance across different areas necessitate active control of artificial lighting. Therefore, it is possible to calculate detailed indoor illuminance in an environment without physical sensors that could affect user activity. This study focused on predicting illuminance at 12 specific points and comparing these predictions with measured values. However, it is also possible to create a more detailed illuminance map, as illustrated in Figure 6c. This study has a limitation: Although it proposes a method for calculating illuminance in a sensorless environment, sensors must be installed during the data collection phase, and if the experimental environment changes, new data must be collected. Therefore, further research is needed to improve the generalizability of the proposed method and ensure its robust performance under varying experimental conditions. Additionally, when applied to real indoor settings, performance can be enhanced by selecting high-quality cameras and optimizing settings such as gain and white balance. This could enable the system to evolve into a lighting control solution that responds to real-time changes in natural light.

## 5. Conclusions

In lighting systems that control illumination in response to changes in light levels, accurate analysis of indoor lighting conditions is essential. However, current methods face challenges of increased costs and inconvenience associated with installing and operating multiple sensors, which hinder the widespread adoption and advancement of related technologies. Therefore, this study proposes a method for generating an image-based indoor illumination map that enables accurate analysis of indoor lighting conditions without requiring the installation of light-measurement sensors in living spaces. An experimental environment was created by constructing a camera module designed to capture images of the indoor floor surface, along with an optical sensing device to gather illuminance data from different indoor areas. These devices were calibrated using a spectroradiometer before being installed. In this experimental setting, an indoor light dataset was created by collecting illuminance and floor-surface images affected by natural light. The correlation analysis results showed a significant association between changes in illuminance and the color elements of indoor (floor surface) images. Subsequently, an initial CNN-based deep learning model was constructed to compute illuminance at each point by feeding each color component of the indoor image. Based on comparative experiments conducted on two selected days, HSV was identified as the optimal input parameter for the proposed method. Furthermore, through hyperparameter tuning, the deep learning model that estimated image-based illuminance was optimized to comprise four Convolutional Layers with 64, 128, 256, and 512 filters, respectively, and Dense Layers with 256 and 96 units. The illuminance values at points P1 through P12 were calculated through an optimized CNN model. The illuminance values were then matched to the indoor space and reconstructed to generate an illuminance map for the 12 indoor points. Through this process, we proposed a method for generating an indoor illuminance map that accurately reflects the constantly changing light environment resulting from the influx of natural light. A performance evaluation experiment was conducted to assess the proposed model on a randomly selected day. The results demonstrated that the model can accurately calculate indoor illuminance at specific points, achieving an MAPE of 11%. Notably, during daytime, when substantial fluctuations in indoor illuminance require active control of artificial lighting, the system attained a high level of accuracy, with a MAPE of just 6%. The study confirmed that it is possible to generate point-specific illuminance data and detailed illuminance maps using only indoor video imagery, without the need for physical illuminance sensors.

In the future, the performance of the proposed method, through the preprocessing of indoor floor images and experiments on combinations of color elements, will be improved to derive optimal input elements for the proposed model. Furthermore, to commercialize the proposed method, experiments are planned in a broader range of environments, including large indoor spaces and offices. Efforts will also focus on developing a sensorless, natural light-integrated lighting system that improves indoor light quality and achieves energy savings by applying the proposed technology.

## Figures and Tables

**Table 1:** Indoor image and illuminance dataset.

Time	T	Illuminance (Lux)/Position (P1~P12)												
	Image	P1	P2	P3	P4	P5	P6	P7	P8	P9	P10	P11	P12	Avg.
07:15	Figure 2a	61	137	132	47	59	54	32	30	36	46	70	64	64
09:00	Figure 2b	380	761	1123	306	393	414	219	216	276	244	366	431	427
12:00	Figure 2c	1314	2768	2150	1062	1330	1205	753	752	839	760	1163	1058	1263
…	…	…	…	…	…	…	…	…	…	…	…	…	…	…

**Table 2:** Correlation Analysis of Color Model Attributes and Illuminance.

Color Model	RGB			Gray-Scale	HSV			LAB			HSL		
(Factors)	(R)	(G)	(B)		(H)	(S)	(V)	(L)	(A)	(B)	(H)	(S)	(L)
Correlation Coefficient	0.61	0.62	0.58	0.62	−0.86	−0.41	0.62	0.61	−0.79	0.9	−0.86	0.62	0

**Table 3:** Illuminance estimation using CNN models: Comparison of results across various color spaces (7 April 2024).

Category Color Space	RMSE	MAE	$R^{2}$
	(Lux)	(Lux)	
RGB	22.898	15.467	0.989
Grayscale	26.270	19.180	0.985
HSV	20.948	15.030	0.99
LAB	30.391	21.343	0.98

**Table 4:** Hyperparameter tuning results.

Rank	Convolution Layers		Dense Layers		MAE (Lux)
	Number of Conv Layers	Filters per Conv Layer	Number of Dense Layers	Units per Dense Layer	
1	4	[64, 128, 256, 512]	2	[256, 96]	13.53
2	5	[64, 256, 512, 512, 512]	3	[192, 32, 64]	13.55
3	4	[128, 128, 384, 512]	3	[256, 64, 96]	13.73
4	5	[64, 256, 384, 512, 512]	2	[128, 64]	14.15
5	5	[96, 256, 512, 512, 512]	2	[192, 96]	14.26

**Table 5:** Results of applying the proposed method.

Category	Illuminance (Lux)											
Time	9:00			13:00			15:21			18:00		
Position	Measurement	Calculation	Error	Measurement	Calculation	Error	Measurement	Calculation	Error	Measurement	Calculation	Error
P1	43	52	9	277	267	10	613	610	3	120	139	18
P2	65	76	11	388	377	11	728	683	45	169	155	14
P3	48	59	11	275	282	7	452	404	49	109	104	5
P4	33	40	7	236	214	22	473	453	21	119	120	1
P5	42	50	8	288	262	26	495	459	35	141	130	11
P6	33	38	5	206	191	15	340	293	48	93	81	12
P7	26	30	5	186	168	17	350	339	11	99	89	9
P8	33	38	6	226	205	21	384	369	15	115	98	17
P9	29	37	8	197	186	11	299	280	19	93	88	5
P10	21	26	5	157	139	18	279	279	0	87	74	13
P11	21	26	5	138	129	9	239	236	3	72	61	11
P12	22	27	5	144	137	7	236	225	11	74	63	11
Average	MAE (MAPE)		7 (20%)	MAE (MAPE)		14 (6.8%)	MAE (MAPE)		22 (5.2%)	MAE (MAPE)		11 (10.45%)
	Total MAPE 11.0% (daytime (13:00, 15:21) average 6%)											

## Data Availability

The datasets presented in this article are not readily available because the data are part of an ongoing study and due to technical/time limitations.
